# Constrained relationship agency as the risk factor for intimate partner violence in different models of transactional sex

**DOI:** 10.2989/16085906.2017.1345768

**Published:** 2017-12

**Authors:** Rebecca Fielding-Miller, Kristin Dunkle

**Affiliations:** 1 Department of Medicine, University of California, San Diego, California, USA; 2 Center on Gender Equity and Health, University of California, San Diego, California, USA; 3 South African Medical Research Council, Pretoria, South Africa

**Keywords:** gender-based violence, intimate partner violence, structural equation model, Swaziland, transactional sex, women’s empowerment

## Abstract

Women who engage in transactional sex are more likely to experience intimate partner violence (IPV) and are at higher risk of HIV. However, women engage in transactional sex for a variety of reasons and the precise mechanism linking transactional sex and IPV is not fully understood. We conducted a behavioural survey with a cross-sectional sample of 401 women attending 1 rural and 1 urban public antenatal clinic in Swaziland between February and June 2014. We used structural equation modelling to identify and measure constrained relationship agency (CRA) as a latent variable, and then tested the hypothesis that CRA plays a significant role in the pathway between IPV and transactional sex. After controlling for CRA, receiving more material goods from a sexual partner was not associated with higher levels of physical or sexual IPV and was protective against emotional IPV. CRA was the single largest predictor of IPV, and more education was associated with decreased levels of constrained relationship agency. Policies and interventions that target transactional sex as a driver of IPV and HIV may be more successful if they instead target the broader social landscape that constrains women’s agency and drives the harmful aspects of transactional sex.

## Introduction

Transactional sex has received a great deal of attention as a driver of HIV and gender-based violence (GBV) in sub-Saharan Africa in the past two decades. While the initial impetus behind this focus was laudable — an epidemiological need to differentiate “informal” sexual exchange from “formal” sex work — the focus in intervention and international circles appears to have drifted over time from a necessary specification of a particular relationship type to something of a moral panic over “vulnerable victims” and their predatory, exploitative male partners (Stoebenau, Heise, Wamoyi, & Bobrova, 2016). This may in part reflect a certain level of societal discomfort with women’s sexuality that does not conform to gender normative patterns (which is to say, submissive and sexually available to a single partner (Schippers, 2007)). Transactional sex in sub-Saharan Africa covers a wide and complex range of relationship types and sexual practices (Stoebenau et al., 2016), but it can best be understood as the informal exchange of sex for money or material support. While distinct from sex work in that neither party typically considers the relationship a commercial exchange (Stoebenau et al., 2016), transactional sex relationships that are operationalised as “the exchange of sex for money or material goods” may double women’s risk of HIV in sub-Saharan Africa (Wamoyi, Stobeanau, Bobrova, Abramsky, & Watts, 2016), and has been significantly associated with heightened intimate partner violence (IPV) and GBV in cross-sectional studies (Dunkle et al., 2007; Dunkle et al., 2006; Jewkes, Morrell, Sikweyiya, Dunkle, & Penn-Kekana, 2012; Jewkes, Sikweyiya, Morrell, & Dunkle, 2011).

The association between IPV and transactional sex is complex. Women’s economic dependence on their male partners may make exiting a violent or exploitative relationship more difficult (Stoebenau et al., 2016). However, in much of the world the male provider role is both normative within heterosexual sexual and romantic relationships and an expected means of enacting hegemonic masculinity — the most culturally powerful way of being a man (Brinig, 1990; Connell, 1987; Glyde, 2016; Wentzell, 2014). Hegemonic masculinity is often asserted and defended through GBV: In South Africa men who endorsed the male provider role within their sexual and romantic relationships were more likely to report perpetrating sexual and physical GBV, had less gender equitable attitudes, and reported more violent behaviour in general (Jewkes et al., 2012). In Tanzania men have reported feeling that they were “forced” to sexually assault women for whom they have provided material support to protect their reputations and assert their masculinity (Maganja, Maman, Groves, & Mbwambo, 2007). Similarly, bystanders may be less likely to intervene in an impending sexual assault if they believe that a man has “bought” the right to have sex with a woman by purchasing her alcohol (Watt et al., 2012).

For many women, gifts and financial support are a key way in which male partners demonstrate their affection (Fielding-Miller, Dunkle, Jama-Shai, et al., 2016; Ruark et al., 2014). Moreover, families and friends may chide a young woman whose partner does not provide her with gifts, as this suggests that he does not value her or consider her to be a legitimate potential partner (Groes-Green, 2013; Wamoyi, Fenwick, Urassa, Zaba, & Stones, 2011). While some women do engage in transactional sex for survival purposes (Miller et al., 2011), others do so to access fashionable consumer goods (Masvawure, 2010; Stoebenau et al., 2013), to support their extended families (Groes-Green, 2013) or primary romantic partners (Cole, 2004), or to cement a long-term relationship (Fielding-Miller, Dunkle, Jama-Shai, et al., 2016). Many of these relationships exist on a spectrum of sexual-economic obligation rather than as a single type of relationship that can be easily assessed with a binary measure. Moreover, many women throughout the world do not explicitly identify their relationships as financially motivated even if they report initiating or remaining in relationships longer than they otherwise would if they were not financially dependent (Dunkle, Wingood, Camp, & Diclemente, 2010). Because of this, transactional sex is likely better measured using a scale of concrete behaviours (i.e., items received and item value) rather than as a subjective binary measure that asks women to identify financial motives as a primary reason for engaging in a sexual relationship (Fielding-Miller, Dunkle, Cooper, Windle, & Hadley, 2016).

Recent research suggests that the mechanism driving the link between transactional sex and HIV is likely more complex than a simple exchange of material goods for sex (Fielding-Miller, Dunkle, Hadley, Cooper, & Windle, 2017; Ranganathan et al., 2016; Wamoyi et al., 2016), and the social and relationship level dynamics that drive the association between transactional sex and IPV are even less clear. This study used structural equation modelling to test: (1) whether different motives for engaging in transactional sex are associated with differing levels of IPV; (2) to what degree the level of transaction within a relationship — measured using a concrete, behavioural scale — affects IPV risk; and (3) the significance and magnitude of women’s agency as a potential mechanism in the link between transactional sex and IPV. Structural equation modelling (SEM) allows the researcher to create a hypothesised set of pathways *a priori* based on theoretical considerations drawn from the literature, and then test how well the theorised model fits the data (Chin, 1998). Our hypothesised structural model was primarily based on what Stoebenau et al. (2016) have labelled the “vulnerable victim” paradigm, in which the link between transactional sex and IPV hinges on women’s economic dependence on their partners, or their place as vulnerable victims within a patriarchal society. This paradigm is particularly dominant among non-governmental organisations (NGOs) and donors, who frequently have the ability to influence policy and programming priorities through funding priorities (Stoebenau et al., 2016).

## Methods

### Study setting and context

The study reported here is part of a larger, mixed-methods research project designed to understand how women in Swaziland conceptualise transactional sexual relationships and how these relationships could best be measured to assess epidemiological associations with HIV and IPV. The study was conducted between November 2013 and June 2014 in the Hhohho and Manzini regions of the country. Data used in this study are from a cross-sectional sample of public antenatal clinic attendees and were collected between February and June 2014.

Swaziland is a small nation in sub-Saharan Africa with a population of approximately 1.1 million (CSO, 2007). One in 3 Swazi adults aged 18–49 are currently living with HIV. The epidemic is highly gendered: the prevalence is 24% among adult men and 39% among adult women, peaking at 54% among women aged 30–34 (Bicego et al., 2013). In Swaziland, as in the rest of the world, women engage in relationships that combine sexual and economic obligation for a wide variety of reasons, including personal pleasure. The Swaziland National Emergency Response Council on HIV/AIDS (NERCHA) has identified both transactional sex and GBV as key drivers of the HIV/AIDS epidemic in the country, and called for more data on social and structural drivers of GBV in the Kingdom (NERCHA, 2014). Both GBV and IPV are likely widespread in Swaziland. There is little population-based data available on the exact scope, however, 1 in 3 young Swazi women have experienced sexual violence before the age of 18 (Reza et al., 2009). In addition 11% of Swazi women report experiencing forced sex in their lifetime (Tsai et al., 2011), although the number may be much higher (Ruark & Fielding-Miller, 2016). Swaziland has high rates of both economic and gender inequality (UNDP, 2015; Southern Africa Regional Resource Centre, 2013), which may exacerbate the risk of transactional relationships (Hickel, 2012).

The parent study used cultural consensus modelling (CCM) to derive emic scales of transactional sex in Swaziland, based on Swazi women’s understanding and valuation of sexual-economic exchange. In CCM the researcher asks a small but statistically significant sample of informants to provide information on how people in their community — rather than themselves personally — perceive or value a certain topic. The researcher then uses principle factor analysis to identify clusters of similar answers. CCM rests on the assumption that if a statistically significant cluster of similar answers exists, it is likely because this is the culturally correct answer (for more details on the CCM process, see Romney, Weller, & Batchelder, 1986; Weller & Romney, 1988). To derive transactional sex CCMs, we first conducted brief free-listing interviews with a convenience sample of Swazi women recruited in public spaces and asked, “What do women in your community receive or hope to receive from a partner when they have sex?” After condensing these answers into a single master list, we then recruited a second convenience sample and asked these women how they believed women in their community would rate the importance of each item from 1–5. We used cultural consensus analysis to identify clusters of women who rated items in similar ways (Weller, 2007) and found three statistically significant clusters of item valuation, which represented three distinct cultural models of transactional sex. The first group, which we labelled “marriage”, is typified by women who are more likely to be married, are slightly older, and are more likely to be living in rural areas. While the prototypical marriage relationship may or may not involve actual marriage, the relationships are usually visible and socially acceptable. Gender roles within marriage style relationships are fairly traditional and normative with patriarchal Swazi culture. In the second group, which we labelled “aspirational”, women were more likely to be living in urban areas and to have completed secondary school. In qualitative interviews, women in aspirational model relationships were typically not married to their partner, although they often tried to position the relationship as long term for social respectability. Partners were expected to provide financial support to demonstrate their affection or to compensate for not being able to provide long-term stability through marriage. The third group consisted primarily of women who had been recruited from a university campus, and so we labelled this group “university”. Women who valued items in a way that was consistent with the university pattern were more likely to articulate a desire for somewhat egalitarian relationships in which gift giving was nominally not mandatory, but was an important way through which their partner demonstrated affection. Qualitative analyses of these relationship models suggest that the borders are both fuzzy and porous, and a woman may move through several relationship models across her life course. Further details on the process of identifying the different models is available at (Fielding-Miller, Dunkle, Cooper, et al., 2016), and a longer explanation on qualitative differences between groups can be found at (Fielding-Miller, Dunkle, Jama-Shai, et al., 2016).

### Study participants

Women were recruited from two public antenatal clinics, one rural and one urban. A female Swazi research assistant (RA) or the first author approached every woman waiting for antenatal services during open clinic hours on recruitment days (generally Monday to Friday) and asked if they were interested in participating in a study. Participants were eligible to participate if they were over 18, comfortable with a survey administered in siSwati, and were receiving antenatal care that day. Antenatal clinic attendees were recruited as HIV was an endpoint for the broader study and all women who attend public antenatal clinics in Swaziland receive an HIV test at every appointment. In Swaziland, approximately 95% of women give birth to at least 1 child in their lifetime and 97% of those attend at least 1 antenatal appointment at some point in their pregnancy (Swaziland Ministry of Health, 2011; CSO, 2007).

### Ethical considerations

The study was reviewed and approved by the Swaziland Scientific and Ethics Committee (SEC) and the Emory University Institutional Review Board. The first author requested and received permission from leadership staff at each clinic and from traditional leadership at the rural study site. Per SEC preferences, monetary incentives were not offered but all participants were provided with refreshments while the survey was administered; childcare was available as necessary. Participants provided written informed consent and were told that they could opt out of the study at any time. At the conclusion of the study, preliminary results were shared with NERCHA and with each of the clinics that had hosted data collection. Results were also shared at a national research conference held in Swaziland in July 2016.

### Measures

The survey was created in English, translated into siSwati, and back-translated into English to check accuracy. It was then pilot tested with a small sample of urban clinic attendees, using cognitive interviewing techniques and modified as necessary to ensure that the intent and translation of each item was clear. A young bilingual female Swazi RA who was familiar with the research project recorded the survey script in siSwati. It was then self-administered by participants using audio computer-assisted self-interview (ACASI) software on laptops. An RA assisted each participant with the basic demographic questions to ensure they were comfortable with the ACASI procedures, and was available to provide assistance on request.

#### Primary predictor: transactional sex scale

Survey participants were shown a list of 22 items generated during the earlier free-listing phase and asked to mark every item that they had received from their most recent sexual partner in the last 12 months. Each item had three different weights, one aligned with the marriage group, one aligned with the aspirational group, and one aligned with the university group (Table 1). For each participant we constructed three transactional sex consonance scores to designate the value of the items received within each model. Because the highest possible total score ranged from 44.4 (marriage) to 70.8 (aspirational), the weighted consonance score was converted to *Z*-scores for comparability. Every participant was assigned a score for each of the three scales so that each woman’s consonance with the marriage, aspirational, and university groups could be assessed separately.

#### Primary outcome: intimate partner violence

We measured emotional, physical, and sexual IPV separately. Participants were asked if in the last 12 months their most recent sexual partner had insulted, intimidated, threatened, slapped, pushed, shoved, hit, thrown something at them, or forced them to have sex when they did not want to do so. Response options were “never,” “once,” “a few times,” and “many times”. Participants who indicated that their partner had insulted, intimidated, or threatened them 2 or more times in the past 12 months were coded as experiencing emotional IPV. Participants who reported being slapped, pushed, shoved, hit, or having something thrown at them 2 times or more in the past 12 months were coded as experiencing physical IPV. Participants who reported any incidents of forced sex from their partner were considered to have experienced sexual IPV.

#### Constrained relationship agency

During the free-listing phase of the research a convenience sample of Swazi women had also been asked to name all the reasons why “Swazi women in your community agree to have sex with a man”. Sample answers included love, sexual satisfaction, marriage, to show commitment, to have children, and to prevent a partner’s infidelity. Antenatal survey participants were shown these items and asked to check all of the reasons why they had agreed to have sex with their most recent partner. Preliminary exploratory factor analysis suggested that poverty, money, hunger, fear a partner would leave, fear of violence, or being forced by parents likely all formed a single latent variable which we labelled “constrained relationship agency” (CRA).

#### Personal and partner variables

Demographic and personal measures included age, residential area, education level, number of children, month of pregnancy, and whether or not their partner drank alcohol. To better understand women’s history of sexual violence participants were asked to categorise their first sexual experience as “willing”, “persuaded”, “tricked”, “forced”, or “rape”. Women who indicated that they were willing or persuaded were collapsed into one category and those who marked tricked, forced, or raped were categorised in a second, coded as “non-consensual first sex” (further details on this decision and the context can be found elsewhere at Ruark & Fielding-Miller (2016).

### Analyses

We first conducted bivariate analyses to compare variables across transactional sex models and by experience of IPV. In the initial bivariate analyses women were assigned to the transactional sex model with which they were the most consonant (i.e., those whose aspirational *Z*-score was highest, indicating that the items they had received were most valued by women in the aspirational transactional sex cultural model, were coded as aspirational; those who scored highest in the university group were coded as university; and those whose consonance *Z*-score was highest for marriage were coded as marriage). We then assessed the unadjusted odds of experiencing each form of IPV by consonance score with each transactional sex model, by CRA indicators, and across demographic and relationship variables.

We next tested whether the six hypothesised reasons for agreeing to have sex indicated a single latent CRA variable by building a confirmatory measurement model. Finally, we constructed a full structural equation model to test the CRA role in the relationship between transactional sex consonance and each form of IPV. To see if this relationship differed by transactional sex model, we ran the full models three times for each form of IPV (for a total of nine models in all), first using each participant’s marriage transactional sex consonance score, then using their aspirational score, and finally based on their university score.

**Figure 1:**
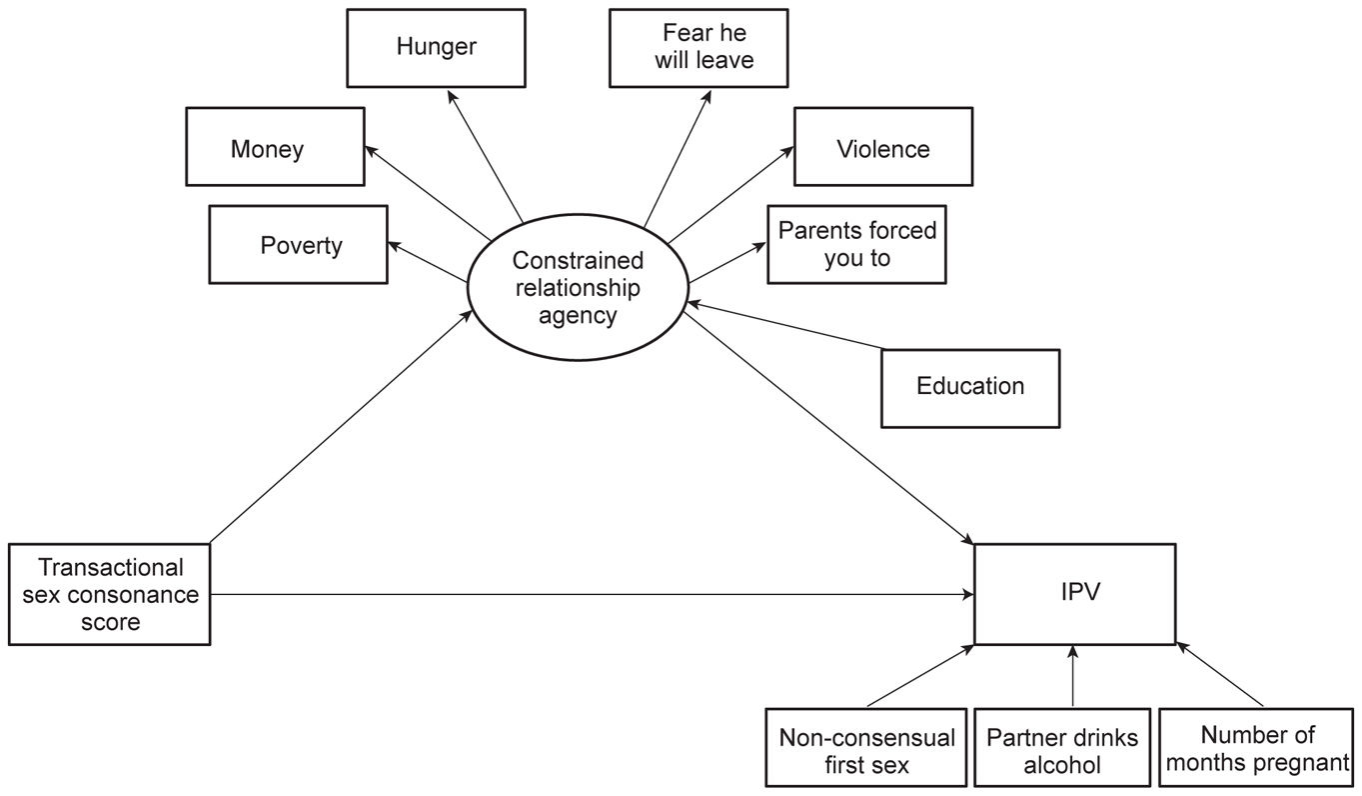
Hypothesised structural equation model

Our hypothesised structural model (Figure 1), theorises that transactional sex leads to constrained agency (per the paradigm that women’s economic dependence may leave them “trapped” in relationships they would otherwise avoid or end (Stoebenau et al., 2016)) and that this lack of ability to exit leads to IPV risk. The model has 20 free parameters and 43 degrees of freedom. While there is some debate over ideal sample size for structural equation models, RMSEA-based criteria (MacCallum, Browne, & Sugawara, 1996) suggest that a model powered at α = 0.05 and β = 0.80 with 43 degrees of freedom, a hypothesised null RMSEA of 0.05, and an alternate RMSEA of 0.08, would require a sample size of 239. Kline (2005) suggests that 200 is a “typical” sample size and that a minimum of 10 participants per parameter is necessary with an ideal ratio of 20 participants per parameter, for a suggested sample size of 400. Global fit for the measurement and structural equation models were evaluated using chi-square tests for “badness of fit”, the Root Mean Square Error of Approximation (RMSEA), and the Comparative Fit Index (CFI), where an insignificant chi-square test, an RMSEA value below 0.06, and a CFI value greater than 0.95 were considered indicators of good overall model fit (Hooper, Coughlan, & Mullen, 2008). Bivariate analyses were conducted using Stata 14 (StataCorp, 2015); measurement and structural equation models were built and tested using the MPlus software package (Muthén & Muthén, 2008).

## Results

### Sample

Approximately 54% of women approached in the clinics agreed to participate. Most of those who declined said they were too busy or did not feel like participating in a study at the time. In all, 406 women completed the survey: 401 (98.8%) provided information on items their most recent sexual partner had provided, 392 (96.6%) completed all questions relating to reasons they had agreed to have sex with their partner, and 403 (95.8%) provided information on IPV experiences. Because <5% of data was missing for all key variables, we used listwise deletion under the assumption that missing data were missing at random (Enders & Gottschall, 2011). All 401 women were included in the confirmatory measurement model to determine if CRA was a valid latent variable. After listwise deletion, a total of 382 women were included in the full structural equation models.

The average participant was approximately 25 years old and in her 6th month of pregnancy with 1–2 children already living in the home (range: 0–11) (Table 2). Based on the items women reported receiving from their partners and the value of those items within different models of transactional sex, 39% of participants (*n* = 156) were most consonant with the aspirational model, 34% were most consonant with the marriage model (*n* = 135), and 27% were most consonant with the university model (*n* = 110). Women who were most consonant with the marriage group may or may not be actually married, however, the items they received from their partner and the way they valued those items was aligned with women who were more likely to be older, rural, and married. As we had found previously in the process of scale development and qualitative exploration, age, education, and number of children varied significantly depending on the model with which women were most consonant. There was no significant difference in rural or urban residency across groups, although the trend was consistent with our previous findings (Fielding-Miller, Dunkle, Cooper, et al., 2016).

Over a third of women reported that their first sexual experience was non-consensual, with a significant difference in prevalence across groups (*p* = 0.03). Women most consonant with the aspirational model were least likely to report that first sex was rape or coercion (28.95%), while women who were most consonant with the marriage model were much more likely to do so (43.05%).

Forty per cent of women reported experiencing some form of IPV in the past 12 months. Emotional IPV was the most common — just over 1 in 4 women reported that their most recent sexual partner had insulted, intimidated, or threatened them more than once — and it differed significantly across groups. Emotional IPV prevalence was highest among women who were most consonant with the marriage model (36.30%), and lowest among women who received items that were most consonant with the university model (20.91%). Approximately 15% of women reported experiencing physical IPV and just under 14% reported sexual IPV, with no significant difference across groups. Ten per cent of women reported experiencing both physical and emotional IPV, 7% both emotional and sexual IPV, and 5% reported physical and sexual IPV. Four per cent of women reported experiencing all forms of IPV in the past 12 months (results not shown).

In unadjusted logistic regression models (Table 3), the amount and value of items received from a partner neither increased nor decreased a women’s probability of experiencing any form of IPV. The sole exception was that for all women, receiving more items that were valued more highly according to the university model was associated with a lower prevalence of emotional IPV (OR: 0.78, 95% CI: 0.61–0.98). Reporting agreeing to have sex with a partner because of poverty or hunger was associated with significantly higher odds of all forms of IPV, although women who reported that they had agreed to have sex with a partner because they feared violence were no more likely to report emotional or physical IPV than women who did not report this as a reason. Overall, for each additional indicator of constrained relationship agency reported, women’s odds of experiencing emotional or physical IPV increased by approximately 50% and their odds of experiencing sexual IPV increased by approximately 80%.

### Latent variable measurement model

Agreeing to have sex with a partner because of poverty, money, hunger, fear he will leave, violence, or because parents forced you to all appear to measure a single latent variable (Table 4). The hypothesised CRA latent variable had excellent global fit, with RMSEA = 0.01, CFI = 0.998, and an insignificant chi-square test (*p* = 0.42) with χ^2^ = 9.27 and 9 degrees of freedom. All of the indicators loaded at *p* < 0.01 with standardised estimates ranging from 0.61 (money) to 0.95 (hunger).

### Structural equation models

Age, number of children, and rural residence were omitted from the final structural models because none were significantly correlated with IPV and our qualitative findings suggested that these three factors were all proxies for the different transactional sex models. Education and month of pregnancy were retained despite their lack of significant correlation in bivariate analyses for theoretical reasons. The results of all three models with standardised coefficients are shown in Table 5. All models had excellent global fit with RMSEA < 0.06, CFI > 0.95, and insignificant chi-square tests except for the model testing the association between consonance with the university model and emotional IPV, which had a chi-square test value of *p* = 0.05 but otherwise excellent overall fit indices.

After controlling for constrained relationship agency and demographic and relationship variables, there was no significant difference in the association between IPV and transactional sex consonance across different models of transactional sex. As in the unadjusted logistic regression models, there did appear to be a trend in which consonance with the university model was more protective against emotional IPV and consonance with the marriage model was less protective, but this trend was not significant.

The amount and value of items that a woman received from her sexual partner was not significantly associated with higher levels of IPV after adjusting for CRA, education, partner, and personal factors. In contrast to many previous findings on IPV and transactional sex, women with higher transactional sex consonance scores were less likely to have experienced emotional IPV in the past 12 months.

The single largest predictor of IPV was constrained relationship agency. Across all models the likelihood of experiencing IPV increased by 0.27 standard deviations (emotional IPV) to 0.35 standard deviations (forced sex) for every standard deviation increase in a woman’s reported CRA (*p* < 0.01, all models). Education appeared to be protective against CRA. In all models, every standard deviation increase in education decreased a women’s CRA by approximately 0.20 standard deviations (*p* = 0.01, all models).

Women who reported that their first sexual experience was non-consensual were significantly more likely to report emotional and sexual IPV (*p* = 0.01 and *p* = 0.03 respectively), but not physical IPV. If a woman reported that her partner drank alcohol, she was 0.18 standard deviations more likely to report emotional IPV (*p* = 0.01) and 0.28 standard deviations more likely to report physical IPV (*p* < 0.01), although a partner’s alcohol use appeared to have no association with her likelihood of experiencing forced sex. For each additional month of pregnancy at the time they took the survey, women were 0.18 standard deviations less likely to report experiencing forced sex from their most recent sexual partner in the past 12 months (*p* = 0.03).

## Discussion

Receiving more material support from a sexual partner was not significantly associated with an increase in intimate partner violence after controlling for women’s constrained relationship agency. In fact, receiving more items that were more culturally valuable was associated with decreased emotional IPV. Our findings suggest that the harmful link between transactional sex and IPV, which has been consistently demonstrated in the literature, may in fact be a product of the broader social landscape in which these sexual-economic relationships are situated rather than the simple acts of financial support and/or gift giving.

Our data reflect a convenience, rather than population-based, sample of Swazi women, and so care should be taken when attempting to generalise our findings to the broader population beyond women who attend public antenatal clinics. Additionally, while our model suggests potential causal directions based on theoretical considerations from the literature, our sample was cross-sectional and so we cannot make any conclusions about causality. However, these analyses do represent the first quantitative test of processes that have been explored for many years with qualitative approaches. Our results are consistent with women’s emic experiences of transactional sex as described in much of the ethnographic literature (Swidler & Watkins, 2007; Tawfik & Watkins, 2007), likely because our measurement approach to transactional sex was rooted in a mixed method project designed to develop emic scales of transactional sex based on Swazi women’s own perspectives and priorities.

When we controlled for women’s relationship agency we found that more material support from a partner was not associated with higher levels of IPV and that contrary to nearly all of the previous literature on transactional sex and IPV receiving more goods that were valued more highly was associated with a decreased, rather than increased, risk of emotional IPV. Our qualitative explorations of the three models suggest that women view gift giving as an important mark of love, affection, and serious intention within romantic relationships; other research in the region has demonstrated similar findings (Fielding-Miller, Dunkle, Jama-Shai, et al., 2016; Ruark et al., 2014; Stoebenau et al., 2016). Qualitative work from Swaziland and Rwanda conducted by Ruark et al. (2017) and published in this special issue suggest that women see gift giving as a tangible way through which they can “see” their partner’s love. The decreased levels of emotional IPV in relationships with higher levels of gift giving suggest that this emic perspective on love is valid and should be heeded by researchers and policy makers from the global North.

Rather than a simple exchange of goods, the primary driver of the association between IPV and transactional sex appears to be CRA as indicated by a woman agreeing to have sex with her partner because of poverty, money, hunger, fear he would leave, violence, or because her parents forced her to. Education significantly decreases women’s CRA, suggesting that efforts to ensure more women and girls have consistent access to high quality education have a great deal of potential to reduce IPV and HIV. In Swaziland, primary education is currently free but secondary and tertiary are not and this can present a financial strain for many families. As we see in Whiteside et al’s (2017) work in this special issue, there are also problems with grade repetition throughout the Swazi public education system, particularly for the orphans and vulnerable children who make up nearly half of school-going children (NERCHA, 2014). Interventions that target young vulnerable women may have an outsize effect on reducing IPV and GBV at a population level. While education is not a panacea, and the quality of a woman’s education is as important as the quantity. These findings are consistent with a broad body of evidence demonstrating that increased access to quality education is associated with lower HIV prevalence (Bicego et al., 2013), decreased risk of IPV (McCloskey, Boonzaier, Steinbrenner, & Hunter, 2016), and an overall improvement in health and gender equity at the individual, family, and community level (UNESCO, 2016). Programmes that provide structural support to families and young women in the form of unconditional cash transfers or microfinance have also been shown to reduce sexual risk behaviours (Pettifor et al., 2016), reduce HIV stigma (Tsai, Bangsberg, & Weiser, 2013), and empower communities to collectively reduce IPV and gender-based violence (Kim et al., 2007).

Women with a history of sexual assault and women whose partners drank alcohol had significantly higher rates of IPV in the full structural model, as did women who reported poverty and hunger in the unadjusted logistic regression models. These findings confirm those from previous studies that have suggested that IPV is driven by a complex social landscape of discriminatory gender norms and policies, toxic masculinity, and economic inequality (Jewkes et al., 2015; Jewkes, Nduna, Jama-Shai, Chirwa, & Dunkle, 2016; Mathews, Jewkes, & Abrahams, 2015; McCloskey et al., 2016; Shannon et al., 2012). Disentangling the effects of gendered relationship norms and economic power dynamics from the simple act of gift giving and financial support within sexual relationships can be difficult, as transactional sex is a product of the same social landscape — the notion of the male provider is a cornerstone of hegemonic masculinity and has been shown to be associated with gender-based violence in South Africa (Jewkes et al., 2012; Jewkes et al., 2011). Policy and programme interventions may be more powerful if they address the root causes that shape this social landscape and drive constrained agency and violence, rather than solely targeting the transactional relationships that arise from these same root causes and as a result are themselves correlated with IPV and HIV.

## Conclusion

Our study demonstrated that constrained relationship agency, not transactional sex, was the strongest predictor of all forms of IPV in the past 12 months. The simple act of receiving financial support from a partner does not appear to increase Swazi women’s vulnerability to IPV, and may in fact be associated with decreased emotional partner violence. Circumstances such as poverty, hunger, or family pressure may limit a woman’s ability to exit a violent relationship, or motivate her to initiate a relationship with a man she may otherwise avoid. Interventions designed to target the link between IPV and transactional sex will be most effective if they target the social landscape that constrains women’s agency, rather than whether or not she receives gifts or material support from a male partner.

## Figures and Tables

**Table 1:** Weighted transactional sex consonance score items

Marriage		Aspirational		University	
		
Item	Weight	Item	Weight	Item	Weight
Basic food	3.9	A fun night out	4.5	Money to provide for children	3.9
Alcohol	3.4	Smart phone	4.4	Fashionable clothes	3.7
Smart phone	3.2	Airtime	4.1	A nice lifestyle	3.7
Basic clothes	2.9	Basic clothes	3.9	Smart phone	3.6
Airtime	2.7	Toiletries	3.7	Basic clothes	3.6
Cosmetics	2.6	Money for hair salon	3.7	Rent	3.5
Fashionable clothes	2.5	Meal at a nice restaurant	3.6	Shoes	3.5
Toiletries	2.5	Alcohol	3.6	Money for hair salon	3.3
Money for hair salon	2.4	Basic food	3.6	A job	3.2
Money to provide for child	2.2	Takeout food	3.6	Money to provide for family	3.0
Money to provide for family	2.1	Fashionable clothes	3.5	Car	2.9
A nice lifestyle	1.9	Rent	3.4	Fun night out	2.7
A fun night out	1.9	Shoes	3.4	Basic food	2.6
Rent	1.7	Jewellery	3.3	Jewellery	2.6
Jewellery	1.6	Transportation	3.2	Cosmetics	2.5
Takeout food	1.3	A place to sleep for the night	3.0	Meal at a nice restaurant	2.4
A job	1.3	A nice lifestyle	2.8	Transportation	2.1
A place to sleep for the night	1.2	Cosmetics	2.8	Airtime	2.0
Shoes	1.0	A job	2.2	Toiletries	1.8
Car	0.8	Money to provide for family	2.1	Alcohol	1.2
Transportation	0.7	Money to provide for children	1.2	A place to sleep for the night	0.7
Meal at a nice restaurant	0.6	Car	1.2	Takeout food	0.5
Total possible score	44.4	Total possible score	70.8	Total possible score	59.0

**Table 2:** Sample demographics

	All % (*n*) or Mean (SD)	Marriage *N* = 135 % (*n*) or Mean (SD)	Aspirational *N* = 156 % (*n*) or Mean (SD)	University *N* = 110 % (*n*) or Mean (SD)	*p*-value
Age	24.54 (4.99)	**25.73 (5.14)**	**22.93 (4.34)**	**25.44 (5.04)**	<**0.01**
Number of children at home	1.52(1.39)	**1.81 (1.52)**	**1.14 (1.37)**	**1.75 (1.10)**	<**0.01**
Rural residence	54.11 (217)	57.78 (78)	55.77 (87)	47.27 (52)	0.23
Month of pregnancy	6.26 (1.85)	6.25 (1.89)	6.31 (1.81)	6.25 (1.86)	0.95
Education					
None	3.49 (14)	**5.93 (8)**	**3.85 (6)**	**0.00 (0)**	
Primary	24.44 (98)	**28.15 (38)**	**12.18 (19)**	**37.27(41)**	
Secondary	63.34 (258)	**61.48 (83)**	**72.44 (113)**	**55.36 (62)**	
Tertiary	7.73 (31)	**4.44 (6)**	**11.54 (18)**	**6.36 (7)**	<**0.01**
First sex tricked, forced, or rape	36.76 (141)	**43.08 (56)**	**28.95 (44)**	**40.19 (43)**	**0.03**
Partner drinks alcohol	34.17 (136)	34.59 (46)	39.10 (61)	26.61 (29)	0.11
IPV, past 12 months					
>1 emotional IPV incidents	28.75 (115)	**36.30 (49)**	**27.74 (43)**	**20.91 (23)**	**0.03**
>1 physical IPV incidents	15.25 (61)	16.30 (22)	12.90 (20)	17.27 (19)	0.57
1+ forced sex incidents	13.75 (55)	12.59 (17)	15.48 (24)	12.73 (14)	0.73
Constrained relationship agency indicators (reasons for having sex)					
Poverty	3.51 (14)	3.73 (5)	3.87 (6)	2.73 (3)	0.87
Money	5.76 (23)	5.97 (8)	7.10 (11)	3.64 (4)	0.49
Hunger	4.01 (16)	4.48 (6)	4.52 (7)	2.73 (3)	0.72
Fear he will leave	6.15 (24)	3.79 (5)	7.38 (11)	7.34 (8)	0.38
Violence	3.59 (14)	3.79 (5)	4.03 (6)	2.75 (3)	0.85
Forced by parents	1.28 (5)	2.27 (3)	1.34 (2)	0.00 (0)	0.30

**Table 3:** Unadjusted odds of experiencing IPV in past 12 months

	2+ emotional IPV incidents		2+ physical IPV incidents		1+ forced sex incidents	
		
	OR	95% CI	OR	95% CI	OR	95% CI
Mean transactional sex consonance Z-score						
Marriage	0.84	(0.67–1.05)	0.98	(0.75–1.29)	1.10	(0.83–1.45)
Aspirational	0.81	(0.64–1.02)	0.90	(0.68–1.19)	1.10	(0.84–1.45)
University	**0.78**	**(0.61–0.98)**	0.95	(0.72–1.25)	1.06	(0.80–1.40)
Constrained relationship agency indicators						
Poverty	**4.88**	**(1.60–14.90)**	**4.61**	**(1.54–13.81)**	**5.41**	**(1.80–16.28)**
Money	1.69	(0.71–4.02)	1.63	(0.58–4.56)	**3.15**	**(1.23–8.06)**
Hunger	**4.55**	**(1.61–12.83)**	**4.86**	**(1.74–13.60)**	**3.18**	**(1.06–9.55)**
Fear he will leave	1.88	(0.81–4.37)	1.49	(0.54–4.16)	**2.87**	**(1.12–7.26)**
Violence	1.94	(0.66–5.73)	2.29	(0.69–7.57)	**3.81**	**(1.22–11.84)**
Forced by parents	1.69	(0.28–10.28)	1.39	(0.15–12.62)	1.61	(0.18–14.65)
Total items indicated (range: 0–6)	**1.59**	**(1.16–2.17)**	**1.53**	**(1.11–2.12)**	**1.80**	**(1.30–2.51)**
Age	1.03	(0.98–1.07)	1.01	(0.96–1.06)	0.97	(0.91–1.03)
Months pregnant	1.07	(0.95–1.20)	0.96	(0.83–1.11)	**0.84**	**(0.73–0.98)**
Number of children	1.02	(0.88–1.20)	1.20	(0.99–1.41)	0.90	(0.71–1.13)
Non-consensual first sex	**2.34**	**(1.50–3.67)**	1.38	(0.79–2.40)	1.65	(0.93–2.93)
Partner drinks alcohol	**1.72**	**(1.10–2.69)**	**3.16**	**(1.81–5.52)**	1.35	(0.75–2.41)
Rural residence	0.90	(0.58–1.38)	0.73	(0.43–1.27)	0.79	(0.45–1.40)
Education	0.92	(0.65–1.29)	0.88	(0.58–1.35)	1.17	(0.74–1.85)

**Table 4:** Constrained relationship agency measurement model

*N* = 401		
chi-square, df (*p*-value)	9.27, 9 (0.41)	
RMSEA	0.01	
CFI	0.998	

Indicator	Standardized estimate	*p*-value

In the last 12 months, did you agree to have sex with your most recent partner because of:		
Poverty	0.73	<0.01
Money	0.61	<0.01
Hunger	0.95	<0.01
Fear he will leave	0.62	<0.01
Violence	0.74	<0.01
Parents forced you to	0.80	<0.01

**Table 5:** Full structural equation model (*n* = 381)

	Emotional						Physical						Forced sex					
		
	Marriage		Aspirational		University		Marriage		Aspirational		University		Marriage		Aspirational		University	
Chi-square, df (*p*-value)	57.69, 43 (0.07)		58.80, 43 (0.06)		58.85, 43 (0.05)		54.91, 43 (0.11)		55.71, 43 (0.09)		56.054, 43 (0.09)		54.94, 43 (0.10)		55.75, 43 (0.09)		56.26, 43 (0.08)	
RMSEA	0.03		0.03		0.03		0.03		0.03		0.03		0.03		0.03		0.03	
CFI	0.967		0.965		0.965		0.973		0.971		0.971		0.973		0.970		0.970	

	Std estimate	*p*-value	Std estimate	*p*-value	Std estimate	*p*-value	Std estimate	*p*-value	Std estimate	*p*-value	Std estimate	*p*-value	Std estimate	*p*-value	Std estimate	*p*-value	Std estimate	*p*-value

CRA																		
TS consonance	0.07	0.51	0.08	0.43	0.05	0.63	0.07	0.51	0.08	0.44	0.05	0.64	0.07	0.50	0.08	0.43	0.05	0.63
Education	−0.21	0.01	−0.21	0.01	−0.20	0.01	−0.22	<0.01	−0.22	0.01	−0.22	0.01	−0.20	0.01	−0.20	0.01	−0.19	0.01
IPV																		
CRA	0.27	<0.01	0.27	<0.01	0.27	<0.01	0.32	<0.01	0.31	<0.01	0.32	<0.01	0.35	<0.01	0.35	<0.01	0.35	<0. 01
TS consonance	−0.16	0.04	−0.18	0.02	−0.19	0.01	−0.04	0.65	−0.11	0.27	−0.04	0.64	−0.01	0.88	−0.02	0.79	−0.02	0.85
Partner drinks alcohol	0.18	0.01	0.18	<0.01	0.18	0.01	0.28	<0.01	0.29	<0.01	0.28	<0.01	0.07	0.36	0.07	0.36	0.07	0.35
Non-consensual first sex	0.25	<0.01	0.25	<0.01	0.26	<0.01	0.10	0.19	0.10	0.18	0.10	0.19	0.17	0.03	0.17	0.03	0.17	0.03
Months pregnant	0.08	0.26	0.08	0.26	0.07	0.28	−0.02	0.83	−0.20	0.81	−0.02	0.83	−0.18	0.03	−0.18	0.03	−0.18	0.03

TS: Transactional sex
